# Students’ Perception of Quality of Learning Experience (Structure, Process and Outcome): Discipline Versus Problem Based Medical Curriculum and the Mediation Role of Process Quality

**DOI:** 10.3390/healthcare10081584

**Published:** 2022-08-21

**Authors:** Mu’taman Jarrar, Radwa Bakr Mohamed, Mohammad Al-Bsheish, Waleed Albaker, Arwa Alumran, Ammar K. Alomran

**Affiliations:** 1 Vice Deanship for Quality and Development, College of Medicine, Imam Abdulrahman Bin Faisal University, Dammam 34212, Saudi Arabia; 2 Medical Education Department, King Fahd Hospital of the University, Al-Khobar 34445, Saudi Arabia; 3 Health Management Department, Batterjee Medical College, Jeddah 21442, Saudi Arabia; 4 Al-Nadeem Governmental Hospital, Ministry of Health, Amman 11118, Jordan; 5 Department of Internal Medicine, College of Medicine, Imam Abdulrahman Bin Faisal University, Dammam 34212, Saudi Arabia; 6 Health Information and Management Department, College of Public Health, Imam Abdulrahman Bin Faisal University, Dammam 34212, Saudi Arabia; 7 Department of Orthopedic, College of Medicine, Imam Abdulrahman Bin Faisal University, Dammam 34212, Saudi Arabia

**Keywords:** problem-based curriculum, learning experience, learner-centered learning, quality of curricular structures, processes and outcomes

## Abstract

Problem-based learning (PBL) is now incorporated into the curricula of most medical schools around the world. In comparison to the traditional curriculum, less is known about the influence of the adoption and implementation of a problem-based curriculum on the perceived structures, processes, and outcomes of learning experiences reported by students. The purpose of this study was twofold: (1) to compare the quality of learning experience of students enrolled in traditional discipline-based and problem-based medical curricula and (2) to explore the mediation effect of the process quality between the relationship of the structural quality and students’ perception of learning experience outcomes. Through the distribution of an electronic survey, all 3rd and 4th year medical students enrolled in the discipline-based curriculum and the problem-based curriculum were invited to participate in the study. The students from both curricula completed the Student Experience Survey (SES), which was developed by the National Center for Academic Accreditation and Evaluation. Descriptive statistics, independent sample *t*-test and Hayes Macro regression analysis were used. Students enrolled in the problem-based curriculum had higher perceived support and sufficient advice with higher perceived quality of learning experiences compared with students enrolled in the traditional curriculum, however they reported less enjoyment of their university life. The structural factors (t = 19.83, *p* ≤ 0.001) and process factors (t = 9.21, *p* ≤ 0.001) were associated with an increase in students’ reported outcomes by 0.67 and 0.49, respectively. These findings explain the mechanism by which the structural factors, such as maintaining adequate facilities and support, may help in enhancing the process quality (e.g., learner-centered learning), which in turn can enhance learning experience outcomes.

## 1. Introduction

Problem-based learning (PBL) is a constructive model of teaching and learning in which the learner can be actively engaged in knowledge building rather than the passive reception of knowledge [1]. Over three decades ago, at McMaster University’s medical school in Hamilton, Ontario, PBL was originally introduced as a distinct curricular method [2]. PBL is now incorporated into the curricula of most medical schools around the world. PBL helps in skills development and enhances students’ motivation to learn and interact with each other [3]. Several studies compared between traditional and PBL curricula [4,5,6]. PBL was found to be a popular and preferred learning style that improves clinical outcomes [4,5], knowledge base [5], and the skills of problem solving [4] and critical thinking [7].

A study conducted in Saudi Arabia found that students enrolled in a PBL curriculum had higher knowledge and skills than students enrolled in a traditional curriculum [6]. Traditional lectures tend to emphasize teaching rather than learning, be passive instead of active, and also tend to be knowledge-based [8]. Even though PBL is becoming more universal, it had a mixed effect on teaching effectiveness; McParland et al. (2004) found a significant difference in students’ examination performance when comparing a PBL curriculum to a traditional curriculum [9]. According to Albanese and Mithchell (1993), PBL graduates demonstrated a higher performance in clinical practice, [10] whereas other studies found that PBL curriculum was associated with an increase in the overall quality of the learning experience, but not associated with improvement in students’ examination performance when compared to the traditional curriculum [9]. Further, despite the fact that PBL improves students’ clinical practice skills and their motivation in educational activities, it was not found to be associated with improved student overall satisfaction [11] and was associated with increased prevalence of depression [12]. These inconsistent findings may be explained by inadequate descriptions of learning conditions and lack of theoretical framing [13]. It is crucial to keep in mind that there are many ways to apply PBL curricula and the student experience is influenced by the style of PBL and the training provided to both staff and students. Several factors such as the involvement of students, group work or evaluation, the students’ impression of workload, quality of instruction or of professional practice relevance, and other factors may affect profound learning among students. A theoretical model may be helpful in guiding educational interventions, teaching, and learning. Therefore, we considered the Donabedian Theory [14] to explore students’ perception of the quality of learning experience.

Donabedian Theory is a quality improvement evaluation theory that links structural factors of healthcare settings with the processes and the outcomes of care [14]. In the current literature, this theory was used to develop a model and establish a system of delivering better quality and safer care [15]. In the current study, it is used primarily as a theory of evaluation that focuses on the impact of structures (such as physical facilities available to the students), processes (represented by the students’ perceptions of the actual learning process and support), and outcomes (represented by the students’ own perceptions of their learning outcomes).

Several studies provided evidence of a link between the learning facilities and students achievements [16,17]. However, little is known about the mechanisms of these associations. Further, no study explored the mediation effect of the process attributes between the effects of the structural factors on the perceived student’s learning experience and outcomes in Saudi Arabia. Therefore, this study aims to explore the differences between the perceived structures, processes and outcomes of learning experience reported by students enrolled in a problem-based curriculum as compared to those enrolled in a traditional curriculum. Further, we explore the mediation effect of the processes attributes between the effects of the structural factors on the perceived students’ learning experience and outcomes.

## 2. Methods

### 2.1. Study Design and Settings

A cross-sectional survey was conducted in the College of Medicine-Imam Abdulrahman bin Faisal University (formerly University of Dammam UoD), Dammam, Saudi Arabia. The college adopted PBL in the medical curriculum since the academic year 2012–2013. The former curriculum, which followed a standard preclinical/clinical study pattern, was replaced with one that was more integrated across disciplines, problem-based, themed, and learner-centered. The new curriculum used both horizontal and vertical integration, allowing students to repeat topics as they moved through the curriculum, expanding their knowledge and skills with each stage. The lectures, tutorials, and rotations were structured and delivered in a way that made it easier for students to make connections between different subject areas and themes. PBL was a fundamental component of the new curriculum. Students enrolled in the traditional curriculum and in the revised (problem-based) curriculum were invited to participate in the study.

### 2.2. Data Collection

The survey was conducted online. Data was collected from 3rd and 4th year medical students, to examine the perceived learning experience among students enrolled in the traditional medical curriculum and the problem-based curriculum. The 3rd year medical students were enrolled in the revised (problem-based) curriculum, while the 4th year students belonged to the traditional curriculum. The 3rd and 4th year students were chosen specifically as they were receiving both pre-clinical and clinical courses, which made the variances lower between the groups. Four weeks before the questionnaires were administered, students received an e-mail from the Vice Deanship for Quality and Development informing them of the current survey (survey aims, confidentiality, and voluntary nature of participation). Four weeks before the final exams of each academic year, students received an e-mail from Vice Deanship for Quality and Development including links to sign a consent form for participation and the survey link. Students willing to participate signed the online consent form and were informed about the confidentiality of their responses and the utilization of their responses for research and quality improvement purposes.

### 2.3. Participants

Through the distribution of an electronic survey, all 3rd and 4th year medical students were invited to participate in the study. A total of 762 medical students were invited to participate in the study, of which 529 were enrolled in the traditional curriculum and 233 in the revised curriculum. Based on Krejcie and Morgan, the sample of 260 students can be considered an adequate sample size. However, some students may not participate; therefore, all students were invited to this study.

### 2.4. Instrument

The Students Experience Survey (SES), a questionnaire developed by the National Center for Academic Accreditation and Evaluation, Saudi Arabia, was utilized in this study. The SES includes 20 items that assess the students’ perceptions of their experience in the program. The questionnaire includes items that relate to the structures, processes and outcomes of teaching quality. The resources (materials, facilities, and human) as well as the organizational structure, policies, and procedures reflect the structural quality [18,19]. Student’s perception of the structural factors was measured by 9 items (e.g., student computing facilities are sufficient for their needs). Process quality refers to the student-centeredness and involvement in teaching and learning, and it represents what is done during the teaching and learning processes. Process quality consisted of 5 items (e.g., the orientation week for new students was helpful). The outcome quality reflects the end result of the teaching and learning processes. It consisted of 6 items (e.g., ability to investigate and solve problems). Students were asked to rate their agreement on a 5-point scale (strongly disagree–strongly agree) for the structure, process, and outcome quality.

### 2.5. Data Analysis

Descriptive statistics, independent sample *t*-test and Hayes Macro regression analysis were used. The Statistical Package for Social Sciences (SPSS) version 21 was used to perform these analyses. Independent sample t-test was used to explore the associations of the implementation of the Problem Based curriculum with the students’ perception of structures, processes and outcomes of learning experience. This preliminary analysis compared the perceived structures, processes, and outcomes among the students enrolled in the problem based curriculum and those enrolled in the traditional curriculum. Eta square and Cohen’s d were performed to identify the magnitude of differences and the effect size. Hayes Macro regression was performed to explore the associations between the changes in the structural and process quality and students’ perceptions of learning experience. The utilization of Hayes Macro aimed at exploring the mediation effect of teaching process factors between the relationship of structural factors and the outcomes. Hayes macro is powerful compared with Baron and Kenny or Sobel’s methods [20,21,22]. Hayes introduced the concept of relative indirect effect (a × b paths) for examining mediation [20,21,22]. The study used 5000 sample Bootstraps. A value of *p* ≤ 0.05 was considered for the level of significance at a 95% Confidence Interval.

## 3. Findings

### 3.1. Descriptive Statistics

A total of 475 students responded to the survey among these, 275 were enrolled in the traditional curriculum and 200 were enrolled in the revised curriculum, representing a 52.0% and 85.8% response rate, respectively. The overall response rate was 475/762 (62.3%).

A total of 159 (33.5%) participants were male and 316 (66.5%) were female students. Descriptive statistics results compared the perception of students enrolled in the traditional curriculum with the students enrolled in the revised curriculum. The mean values indicated a higher rating of perceived services; facilities and outcomes among students enrolled in the problem-based curriculum compared to students enrolled in the traditional curriculum.

### 3.2. Multivariate Assumbtions, Reliability and Validity

Multivariate assumptions (normality, linearity, homoscedasticity and multicollinearity) and factor analysis of the questionnaire validity were tested. For the purpose of identifying normality, kurtosis and skewness statistical approaches were applied. The findings showed that the skewness and kurtosis were both within two standard deviations, thus normalcy was assumed [23]. Additionally, the scatterplots of the variables’ standardized residuals were examined to further confirm the linearity [24]. The multicollinearity assumption was diagnosed using the tolerance and VIF values [25]. The multicollinearity assumption was not violated because there were no tolerance values below 0.10 and no VIF values beyond 10. When normality is assumed, this means that the relationship between the variables is homoscedastic.

Reliability and validity of the study questionnaire items were examined. Cronbach’s Alpha results indicated a high level of internal consistency for the survey items with a value of 0.939. Furthermore, exploratory factor analysis indicated a valid instrument with KMO (Kaiser–Meyer–Olkin) value of 0.942, and Bartlett’s significance value *p* ≤ 0.001, with high factor loading items (six items of structural quality, five items of process quality and six items of outcome quality). With regard to the factor analysis, the loading factors of items less than 0.50 were omitted from the analysis for greater interpretation of variances that share at least 25.0% of the variability of the construct [23,24]. Three items were omitted with loading factor less than 0.50 (Q1, Q5 and Q8). This does not mean that the omitted items were not important, but that they had little incremental predictive power and their effect was already represented by other included items under the corresponding dimension [24] (See Table 1).

### 3.3. Comparing the Scores of Structure, Process, and Outcome

An independent sample *t*-test was conducted to compare the scores of structural, process, and outcome quality among students enrolled in the traditional curriculum and those enrolled in the problem-based curriculum, as shown in Table 2. As regards the structural factors, there were no significant differences between the students enrolled in the traditional (Mean = 3.25, SD = 0.83) and the PBL curriculum (mean = 3.39, SD = 0.93); at t = −1.65 and *p* = 0.101. The magnitude of differences was very small with Eta squared = 0.006 and small effect size with Cohen’s d = 0.156 (Eta squared < 0.01 and Cohen’s d < 0.2) [25]. Therefore, only 0.6 percent of variances between the students could be explained by structural factors, indicating that facilities and resources used for conducting both curricula were similar.

Regarding the process factors, there were significant differences between the students enrolled in the traditional curriculum (Mean = 3.33, SD = 0.78) and those enrolled in the problem-based curriculum (mean = 3.53, SD = 0.93); at t = −2.54 and *p* ≤ 0.05. Students enrolled in the problem-based curriculum reported higher perceived support and advice. However, the magnitude of differences was small with Eta squared = 0.013, and small effect size with Cohen’s d = 0.231 (Eta squared is close to 0.01 and Cohen’s d is close to 0.2) [25]. Accordingly, only 1.3 percent of variances between the student groups could be explained by process factors.

In terms of the outcomes, there were significant differences between the students enrolled in the traditional curriculum (Mean = 3.60, SD = 0.80) and those enrolled in the problem-based curriculum (mean = 3.85, SD = 0.93); at t = −3.13 and *p* ≤ 0.01. Students enrolled in the problem-based curriculum reported higher perceived outcomes compared with students enrolled in the traditional curriculum. However, Q20 (“Overall, I am enjoying my life as a student”) indicates a lower score for the students enrolled in the PBL curriculum. The magnitude of differences was small with Eta squared = 0.020 and small effect size with Cohen’s d = 0.289. Therefore, several factors were required to be included in order to explore the variances of students’ learning experiences and outcomes.

### 3.4. Structure, Process, and Outcome of Students’ Learning Experience

The Hayes Macro regression results, shown in Table 3, indicate the effect of structural and process factors on the outcome quality and measures the mediation effect of the process factors on the outcomes of the students’ learning experience. The results show that structural factors (B = 0.67, t = 19.83, *p* ≤ 0.001) and process factors (B = 0.49, t = 9.21, *p* ≤ 0.001) have a significant association with the outcomes quality. One unit improvement in the structural and process factors predict the outcomes by 0.67 and 0.49, respectively. Therefore, students’ learning experiences and outcomes can be predicted as a result of changes in the structure and process.

Further, the structural factors (B = 0.78, t = 29.05, *p* ≤ 0.001) predict teaching and learning processes. In other words, students who perceived a superior availability of resources perceived higher student engagement and support. The indirect effect indicates that process factors such as student support and engagement (B = 0.29, t = 5.52, *p* ≤ 0.001), mediate the relationship between the structural factors and the outcomes of medical education. Therefore, supported and engaged students had higher perceived outcomes. The summarized results of F = 273.98, R^2^ and adjusted R^2^ values indicated that the study variables predict 54% of variances in optimizing medical students’ learning experience outcomes.

## 4. Discussion

The findings of this study revealed that students enrolled in the problem-based curriculum had higher perceived support, sufficient advice and a higher quality of learning experience when compared to students enrolled in the traditional curriculum. Students in the problem-based curriculum had higher ratings of their learning experience, processes (e.g., perceived more student-centered learning) and outcomes. These findings were supported by the literature; PBL is a student-centered approach [8] that helps in predicting students’ learning outcomes [26]. However, there were no significant differences between the two study groups as regards the perceived structural quality. These findings were justified by the fact that both groups of learners were using the same facilities in the College of Medicine. Interestingly, the findings of our study revealed that students enrolled in the problem-based curriculum reported higher perceived outcomes compared with students enrolled in the traditional curriculum with the exception of their level of enjoyment of university life. This might support the fact that PBL students are putting significantly more effort to cover their curriculum requirements as compared to the students that follow the traditional curriculum.

Furthermore, the study found that the structural factors and process factors predict the students’ perceived learning outcomes. The availability of adequate facilities and the implementation of student-centered learning techniques can be associated with the outcome quality of the students’ learning experience. Adequate facilities and student-centered learning can predict students’ perceived learning outcomes (e.g., cognitive abilities; problem solving and critical thinking and enhance their abilities such as effective communication and ability to work in groups). These findings were supported by previous studies which confirmed that the quality of school facilities was associated with students’ achievement [16]. For instance, library facilities were shown to help enhance teaching effectiveness [27]. Further, team-based learning and effective communication were shown to help enhance clinical education [28].

The study findings revealed a mediation effect of process quality between the effect of the structural factors on the perceived students’ learning experiences and outcomes. This explains the mechanism by which the associations of the structural factors, such as maintaining adequate facilities and support, help in enhancing learner-centered learning which in turn can enhance the outcomes of teaching. For instance, previous studies found that class size and course planning are associated with the perceived overall satisfaction with clinical teaching among medical students [17]. This indicates that in a low volume class size, students have better opportunities to get a person-centered education which in turn helps in optimizing the outcomes of teaching. Therefore, to reach the ultimate goal of optimizing learning outcomes, it is crucial to consider the structural factors as well as the process factor, to focus on the students’ needs by providing a more person-centered medical education.

### Limitations and Future Directions

There are limitations in the generalizability of the findings of this study, as this is a cross-sectional study that was carried out in one institution, even though the college of medicine in IAU is a public college that accepts students from overall Saudi Arabia. However, future research encompassing several institutions is required to better examine the study model. The study nature also limited our ability to establish the causality of the study model, and future work is required to replicate it using other study designs to achieve a better understanding of the causal associations. Furthermore, the study refers to the analysis of the student feedback on the quality of the learning experience. However, the study lacks the comparison of the actual student performance in specific courses which could be a more objective criterion. Future research is required to compare students’ achievement and the effectiveness of the different learning methods in the study model. Additionally, the study was limited to the structural and process factors affecting student’s experiences and outcomes. Further studies examining the contextual factors (learning environment, faculty support, work condition, workload, management commitment and teaching quality) [29,30,31,32] and student related factors (parents’ education, family income, etc.) are required in the future.

## 5. Conclusions

This study presents students’ perceptions of their learning experience in the problem-based and the traditional curriculum. The comparison involved the students’ perceptions of the structures, processes, and outcomes for students enrolled in traditional and problem-based curricula. Significant differences were found between students’ perceptions of process quality, but not perceptions of structure. Students in the PBL group also reported higher perceived support and better outcomes as compared to traditional learners. The students in the problem based curriculum perceived more opportunities to get advice in their study, course enrolment, and future career, and less enjoyment with their university life in comparison to the students enrolled in the traditional curriculum. Adequate facilities for extracurricular activities, fairness by faculty, and students’ encouragement helped to enhance students’ confidence to investigate and solve problems, communicate, and work in groups more effectively and encouraged further learning.

The findings of the study demonstrated that process quality mediates the effect of structural elements on students’ perceptions of their learning experiences and outcomes. This describes how structural variables, such as providing proper facilities and support, contribute to increase learner-centered learning, which can enhance teaching outcomes. Therefore, this article provides an insight for policy makers about the importance of instilling the culture of student-centered education in order to increase student participation and engagement in the teaching processes. In conclusion, student-centered education as a process factor complements the association between the structural factors on the outcomes of medical education.

### Implications

To our knowledge, this is the first study that compared the perceived structure, process and outcome of students’ learning experience between students enrolled in a traditional, discipline based, medical curriculum and students enrolled in a problem based curriculum in Saudi Arabia. Further, this study explored the mediation effect of the process quality between the effect of the structural factors on the perceived student’s learning experience outcomes. The advantages of this study are providing practical experiences for policy makers.

The proposed theoretical model was developed based on the Donabedian theory of quality improvement [14]. Researchers have used this theory to develop a model and establish a system of delivering better quality and safer care [15,33,34,35,36]. The NCAAA instrument was validated in this study for measuring the theoretical model, which can be used by researchers in future research for identifying gaps and for optimizing and providing an enhanced learning experience

The practical implications were related to the importance of student-centered learning. Students are the focal point of teaching and learning; in view of the complexity of implementing a student-centered learning approach [37], our study provides insights for policy makers on the importance of channeling resources to ensure high quality, accessible facilities for maintaining students’ learning experience in medical education.

## Figures and Tables

**Table 1:** Factor analysis results.

Q#	Items	Structural Quality	Process Quality	Outcome Quality
Q11	Adequate facilities are available at UoD for religious observances.	0.797		
Q10	Adequate facilities are available for extracurricular activities (including sporting and recreational activities).	0.781		
Q6	Student computing facilities are sufficient for my needs.	0.769		
Q14	My courses and assignments encourage me to investigate new ideas and express my own opinions.	0.702		
Q4	Procedures for enrolling in courses are simple and efficient.	0.700		
Q9	The library is open at convenient times.	0.672		
Q3	There is sufficient opportunity at UoD, to get advice on my studies and my future career.		0.797	
Q2	When I first started at UoD, the orientation week for new students was helpful for me.		0.764	
Q12	Most of the faculty with whom I work at UoD are really interested in my progress.		0.731	
Q13	Faculty at UoD are fair in their treatment of students.		0.635	
Q7	The library staff are helpful to me when I need assistance.		0.633	
Q16	My ability to effectively communicate the findings of such investigations is improving as a result of my studies.			0.854
Q18	The knowledge and skills I am learning will be valuable for my future career.			0.832
Q15	As a result of my studies my confidence in my ability to investigate and solve new and unusual problems is increasing.			0.830
Q17	My program of studies is stimulating my interest in further learning.			0.801
Q20	Overall, I am enjoying my life as a student at UoD.			0.760
Q19	I am learning to work effectively in group activities.			0.727
	Variances explained	54.541%	51.121%	64.310%
	Eigenvalue	3.272	2.556	3.859
	KMO	0.834	0.738	0.854
	Bartlett’s (Sig)	≤0.001	≤0.001	≤0.001

**Table 2:** Independent sample *t*-test results comparing the scores of students’ learning experience in the problem-based and the traditional curriculum.

Factor		N	Mean (SD)	Std. Error	t	*p* Value	Eta Squared (Cohen’s d)
Structure Quality	Traditional Curriculum	275	3.25 (0.83)	0.05	−1.65	0.101	0.006 (0.156)
	Revised Curriculum	200	3.39 (0.93)	0.07			
Process Quality	Traditional Curriculum	275	3.33 (0.78)	0.05	−2.54	0.011 *	0.013 (0.231)
	Revised Curriculum	200	3.53 (0.93)	0.07			
Outcome Quality	Traditional Curriculum	275	3.60 (0.80)	0.05	−3.13	0.002 **	0.020 (0.289)
	Revised Curriculum	200	3.85 (0.93)	0.07			

*: Significant at *p* ≤ 0.05, **: Significant at *p* ≤ 0.01.

**Table 3:** Hayes Macro regression analysis results: the relationships between structural, process, and outcome quality.

Variable	B	SE	t	*p*
Structure-Process “a path”	0.78	0.03	29.05	≤0.001
Process-Outcome “b path”	0.49	0.05	9.21	≤0.001
Structure-Outcome “c path”	0.67	0.03	19.83	≤0.001
Structure-Process-Outcome “c’ path”	0.29	0.05	5.52	≤0.001
**Model Summary**				**Value**
R^2^				0.537
Adj R^2^				0.535
F				273.982
Sig				≤0.001

## Data Availability

Data Available upon request from the corresponding author (M.J.).
